# The Action of Alcohol in Acute Disease

**Published:** 1898-01-29

**Authors:** 


### THE ACTION OF ALCOHOL IN ACUTE DISEASE.

In a lecture recently delivered before the British Medical Temperance Association, Dr. Sims Woodhead, in describing some recent researches on the action of alcohol, mentioned those made by Dr. Delearde with the object of ascertaining the effect of giving alcohol to animals in whom artificial immunity was being produced.

We would direct the attention of the profession to the results obtained, because they seem to us to have a very direct bearing on the administration of alcohol in certain acute diseases. In regard to rabies, tetanus, and anthrax, the effect of giving alcohol while the immunising process was being carried on was that the immunisation was very seriously interfered with. In regard to rabies it is said that "little or no immunity had been acquired"; in tetanus that animals so treated "do not readily acquire immunity"; and in anthrax that "it is almost impossible to confer immunity if the animal is alcoholised during the time that it is being vaccinated." We need hardly point out how widespread are the deductions which must be drawn in regard to the use of alcohol in acute infectious disease if these observations are to be accepted as fairly representing its effect on the processes by which, in the course of a malady, immunity is gradually attained, and by which the disease itself is ultimately mastered. If we are to picture to ourselves the natural mode of cure in infectious diseases as arising from a reaction on the part of the leucocytes and tissue cells to the toxins produced by the invading microbes—the production of anti-toxic substances by a vital action on the part of the white and fixed cells—and if alcohol holds this vital action in abeyance, then there would seem to be good scientific reason against the administration of alcohol in such conditions. We have already pointed out, and this only bears the fact in upon us all the more, that the time has come when the action of many medicines and alcohol among them, requires to be investigated entirely afresh, and on a bacteriological basis. Alcohol has been looked upon as an antipyretic, as a vaso-dilator, as a heart tonic, as a nerve stimulant, as a sedative, and as a score of other things; but, after all, in the treatment of acute diseases caused by the invasion of micro-organisms, its real importance must depend upon its action upon the invading microbes, or upon the vital processes by which their invasion is resisted, and everything that throws light upon this is a matter of great interest.

